# Kefir

**Published:** 1887-01

**Authors:** 


KEFIR.—Kefir, or fermented cow's milk, is now quite generally used in European countries. Quite recently a German has undertaken to introduce it into Buffalo, and is now able to furnish it to customers at 25 cents a quart. Theodorff, in *Schmidt's Jahrbuch*, reports a large number of cases of anæmia, phthisis and diarrhœa treated by this agent. The following is a summary of his conclusions.

1. Kefir increases the quantity of urine, and the solids and specific gravity diminish.
2. Metamorphosis of tissue is reduced.
3. Digestion and assimilation improve
4. Weight of the body increases.
5. Red blood corpuscles increase in number.
6. Pains accompanying chronic diseases disappear and sleep is improved.
7. It is especially indicated in convalesence from exhausting diseases. The only contra indications to its use are obesity and a tendency to apoplexy.

---

IN 1880, Buffalo, with a population of 155,000, had 231 physicians, or one to every 700 inhabitants. During the past six years 226 physicians have located in the city, while 13 have died and 98 moved away. There are accordingly in Buffalo at this date, 346 physicians, with a probable population of 212,000, or one physician to every 600 inhabitants. The population has increased 37 per cent. in six years, while the number of physicians has increased 50 per cent. During this time, also, the sanitary condition of the city has been greatly improved and the death rate has fallen from 24 per 1,000 to 19.8 per 1,000, a reduction of 20 per cent., with a corresponding reduction in the amount of sickness. We may therefore conclude that the average physician to-day has not more than two-thirds as much to do as the doctor of 1880, since he has but 87 per cent. as many patrons, and but 80 per cent. of the amount of sickness.

